# Comparison of T Cell Receptor-Induced Proximal Signaling and Downstream Functions in Immortalized and Primary T Cells

**DOI:** 10.1371/journal.pone.0005430

**Published:** 2009-05-04

**Authors:** Rebekah R. Bartelt, Noemi Cruz-Orcutt, Michaela Collins, Jon C. D. Houtman

**Affiliations:** Department of Microbiology, University of Iowa, Iowa City, Iowa, United States of America; New York University School of Medicine, United States of America

## Abstract

**Background:**

Human T cells play an important role in pathogen clearance, but their aberrant activation is also linked to numerous diseases. T cells are activated by the concurrent induction of the T cell receptor (TCR) and one or more costimulatory receptors. The characterization of signaling pathways induced by TCR and/or costimulatory receptor activation is critical, since these pathways are excellent targets for novel therapies for human disease. Although studies using human T cell lines have provided substantial insight into these signaling pathways, no comprehensive, direct comparison of these cell lines to activated peripheral blood T cells (APBTs) has been performed to validate their usefulness as a model of primary T cells.

**Methodology/Principal Findings:**

We used quantitative biochemical techniques to compare the activation of two widely used human T cell lines, Jurkat E6.1 and HuT78 T cells, to APBTs. We found that HuT78 cells were similar to APBTs in proximal TCR-mediated signaling events. In contrast, Jurkat E6.1 cells had significantly increased site-specific phosphorylation of Pyk2, PLCγ1, Vav1, and Erk1/Erk2 and substantially more Ca^2+^ flux compared to HuT78 cells and APBTs. In part, these effects appear to be due to an overexpression of Itk in Jurkat E6.1 cells compared to HuT78 cells and APBTs. Both cell lines differ from APBTs in the expression and function of costimulatory receptors and in the range of cytokines and chemokines released upon TCR and costimulatory receptor activation.

**Conclusions/Significance:**

Both Jurkat E6.1 and HuT78 T cells had distinct similarities and differences compared to APBTs. Both cell lines have advantages and disadvantages, which must be taken into account when choosing them as a model T cell line.

## Introduction

Human T cells control the extent and focus of the adaptive immune response to pathogens. T cells are activated by the interaction of the cell surface, multi-subunit T cell receptor (TCR) with an antigen-bound major histocompatibility complex present on an antigen presenting cell [1], [2]. In addition to TCR induction, T cells also require an activating signal from one or more costimulatory receptors, such as CD28 or the α4β1 integrin VLA-4, to become fully active [1]. Costimulation is critical for the specificity of the immune response because it allows T cells to be activated only during acute infection. This enables the adaptive immune system to mount a response to foreign invaders while tolerating its own cells. The mistaken recognition of self leads to aberrant T cell activation, resulting in numerous human disease, such as autoimmune diseases, cardiovascular disease and allergies/asthma [3], [4], [5]. Signaling pathways that are activated by TCR and/or costimulatory receptors are good targets for the development of therapies to these diseases [4], [5]. However, before effective therapies can be developed, we must first better understand the intracellular signaling that occurs when a T cell is activated.

**Figure 1:**
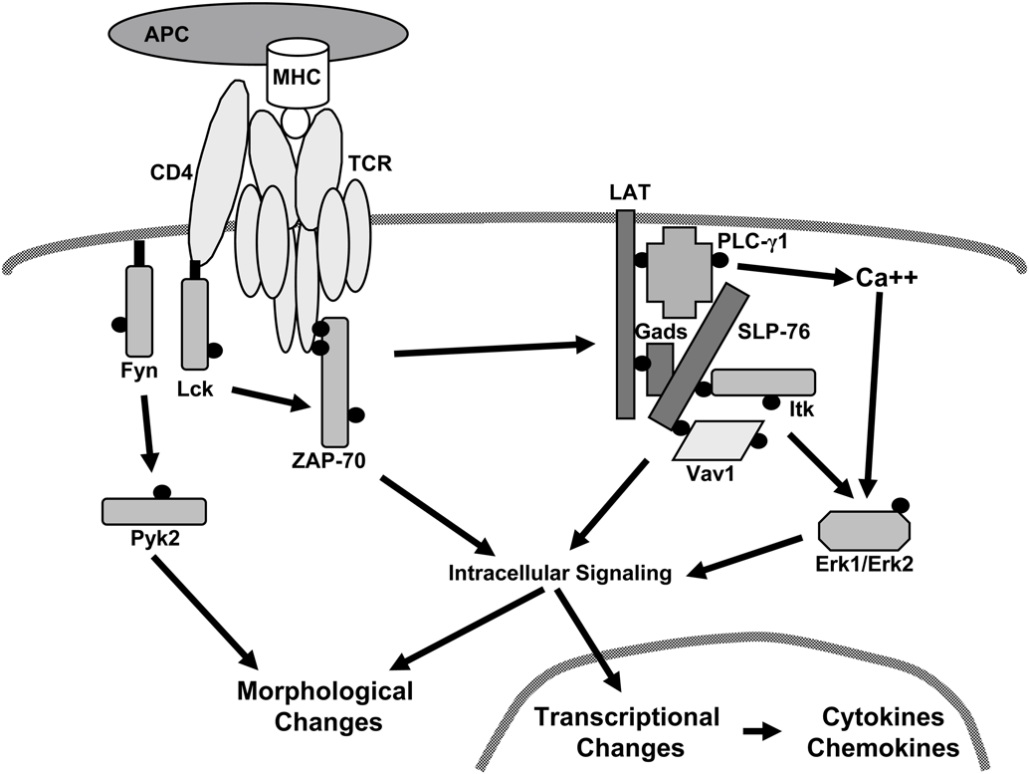
Current model of proximal signaling pathways downstream of TCR activation. TCR activation leads to the induction of numerous tyrosine kinases and adaptor proteins. The activation of these signaling molecules leads to morphological changes and alterations in transcription that are vital for T cell activation and function. Proteins that are tyrosine phosphorylated upon TCR stimulation are identified with small black circles.

An initial event upon TCR activation is the induction of the Src family kinases Lck and Fyn, which then phosphorylate the immunoreceptor tyrosine-based activation motifs (ITAMs) present on several TCR subunits (Figure 1) [1]. The protein tyrosine kinase ZAP-70 is recruited to the phosphorylated ITAMs and activated upon phosphorylation of tyrosine 319 [1]. Activated Lck, Fyn, and ZAP-70 then phosphorylate multiple downstream substrates, including linker for activation of T cells (LAT) and the tyrosine kinase Pyk2 [6], [7], [8]. Pyk2 is a member of the Fak family of kinases and appears to control actin cytoskeletal rearrangements that are critical for T cell activation [6]. LAT is a hematopoietic-specific adaptor protein that mediates many downstream events following TCR stimulation. Upon TCR activation, LAT is phosphorylated on five conserved tyrosines, which then bind to several SH2 domain-containing proteins, such as the related adaptor proteins Grb2, Grap, and Gads, as well as PLC-γ1[8]. Once recruited to LAT, PLC-γ1 is phosphorylated by the Tec family tyrosine kinase Itk at tyrosine 783. This leads to the increased ability of PLC-γ1 to cleave phosphatidlyinositol (4,5) bisphosphate into inositol (1,4,5) trisphosphate, which is important for Ca^2+^ influx, and diacylglycerol, which is important for protein kinase C activation [9].

PLC-γ1 and the Grb2 family of adaptor proteins all contain SH3 domains that mediate the recruitment of signaling proteins to LAT. Once such protein is SLP-76, an adaptor protein that is brought to the LAT complex via its interaction with the adaptor protein Gads and PLC-γ1 (Figure 1) [10]. Upon TCR stimulation, SLP-76 is phosphorylated and both Vav1 and Itk bind to these phosphorylated tyrosines [10]. Vav1 is a guanine nucleotide exchange factor for Rac, a small GTPase that triggers cytoskeletal rearrangements downstream of TCR induction [11]. Vav1 requires the presence of Itk to bind phosphorylated SLP-76, although it does not appear that Vav1 and Itk interact directly [12]. Similarly, Itk requires binding to phosphorylated SLP-76 to maintain its active conformation [13]. The end result of these interactions at the LAT complex is the induction of important downstream functions needed for T cell activation, such as Ca^2+^ flux, Erk1/Erk2 activation, receptor upregulation and the induction of cytokine release.

Much of our understanding of TCR-mediated signaling was discovered using two human T cell lines, the Jurkat E6.1 T cell and the HuT78 T cell. The original Jurkat cells were derived from a 14-year-old boy with T cell acute lymphoblastic leukemia (T-ALL). These cells are thymocytically derived with the characteristics of immature thymocytes. The Jurkat E6.1 subclone cell line was developed in the 1980s [14]. Sublines of Jurkat E6.1 cells have been isolated that are deficient in several TCR signaling molecules, including stable lines lacking the TCRβ chain, Lck, LAT, SLP-76, ZAP-70, Vav1, or PLC-γ1 [14]. These mutants have yielded much information and insight into the function of many peripheral TCR signaling molecules. The HuT78 T cell line is historically a very important cell line: the H9 subclone of HuT78 T cells is the cell used to isolate HIV. HuT78 cells were derived from a Sezary syndrome patient, and are CD4+, mature, cutaneous, lymphoid T cells [15].

Despite being highly useful for several reasons, such as mutant sublines, high rates of transfection, and ease of growth, Jurkat E6.1 cells have several known abnormalities. These include deficiencies in PTEN and SHIP, lipid phosphatases that regulate phosphoinositide-3 kinase (PI3K) function [16], [17]. The lack of PTEN and SHIP leads to several irregularities including constitutive expression of AKT, increased levels of phosphorylated phosphoinositide lipids, and the constitutive association of Itk with the plasma membrane [17], [18]. HuT78 cells also have deficiencies; they have abnormal c-myc function and over express Bcl-xL, suggesting problems with anti-apoptotic pathways in these cells [19], [20].

Although all cell lines have inherent problems, there are many reasons to use cell lines as model systems for studying the activation and function of human T cells. Cell lines are useful because they offer an unlimited, genetically manipulatable and widely accessible supply of experimental material that can be stored long-term and grown at a low cost. Using continuous cell lines also reduces the human burden and ethical considerations of experimentation and substantially reduces donor to donor variation. It is important however, that the pros and cons of a specific cell line are appreciated to allow for interpretation of experiments using these cell lines. Surprisingly, there are no previous studies that directly compare TCR and/or costimulatory-mediated signaling and function in the human T cell lines to human activated peripheral blood T cells (APBTs). Therefore, we chose to compare the Jurkat E6.1 T cells and HuT78 T cells to APBTs in an effort to determine the conduciveness of using these T cell lines as a model for peripheral TCR signaling and function. We found that there was no noteworthy difference among the three cell lines when examining early signaling events such as ZAP-70, LAT, and SLP-76 phosphorylation. However, there were significant differences in downstream signaling events, costimulatory receptor expression and activation and in the cytokines and chemokines released upon TCR induction. Advantages and disadvantages exist for both cell lines, which must be considered during the experimental design process.

## Results

In order to compare proximal TCR-mediated signaling events in Jurkat E6.1 T cells, HuT78 T cells, and APBTs, equal cell numbers were activated with maximal doses of stimulatory TCR antibodies. The samples were then analyzed by immunoblotting using phosphospecific and pan antibodies to specific signaling molecules. We calculated the ratio of phosphorylated protein to total protein for each specific protein using quantified densities from the immunoblots. By using this method, the total phosphorylation per protein of a specific signaling molecule for each cell type was determined, regardless of the size of the cell or the concentration of protein present in each cell. Thus, the relative extent of activation of key signaling molecules was compared between each cell type.

### Jurkat E6.1 T cells, HuT78 T cells, and APBTs had significant differences in the phosphorylation of the tyrosine kinases ZAP-70, but not the adaptor proteins LAT and SLP-76

**Figure 2:**
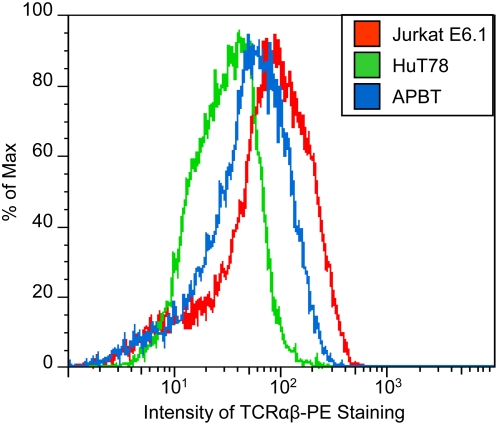
HuT78 T cells have less TCR surface expression than Jurkat E6.1 T cells and APBTs. The surface expression of the TCR α/β chain on Jurkat E6.1 T cells, HuT78 T cells, and APBTs was assessed by flow cytometry. A representative plot of the TCR surface expression of these cell lines is shown. The isotype control for each cell had a mean fluorescent intensity of <8. The experiment was repeated three times.

Before the relative activation of critical signaling molecules can be assessed, the surface expression of the TCR αβ chain in the various T cell lines must be determined. This information is needed because differences in the surface expression of the TCR αβ chain in Jurkat E6.1 T cells, HuT78 T cells and APBTs may result in variations in receptor-mediated signaling events. To this end, the surface expression of the TCR αβ chain in the Jurkat E6.1 T cells, HuT78 T cells and APBTs was examined by flow cytometry. The mean fluorescent intensity (MFI) of the TCR αβ chain expression for three separate experiments was 78+/−19 for Jurkat E6.1 T cells, 29+/−9 for HuT78 T cells and 60+/−8 for the APBTs (Figure 2 and data not shown). It was consistently observed that HuT78 T cells had significantly less TCR αβ chain expression than Jurkat E6.1 T cells and APBTs, whereas there was no significant difference in the expression of TCR αβ chain between the Jurkat E6.1 T cells and APBTs (Figure 2). Thus, theHuT78 T cells have significantly less TCR surface expression than the Jurkat E6.1 T cell and APBTs. However, whether this difference leads to alterations in the extent of intracellular signaling is unknown.

**Figure 3:**
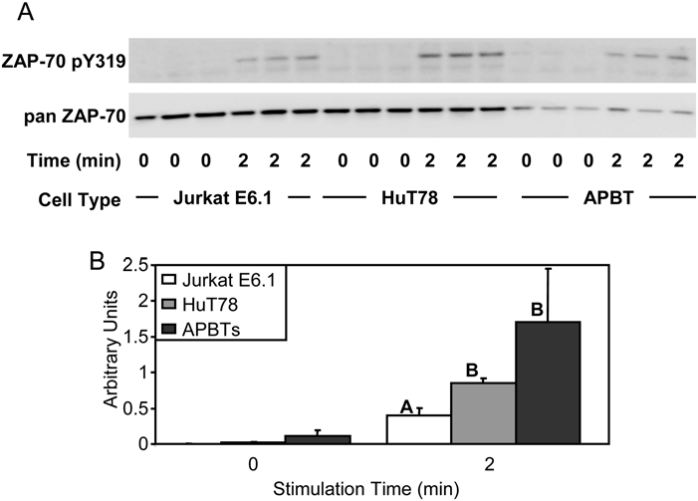
Jurkat E6.1 T cells exhibit decreased ZAP-70 Y319 phosphorylation compared to HuT78 T cells and APBTs. (A) Three samples of Jurkat E6.1 T cells, HuT78 T cells, and APBTs were stimulated and the cellular proteins were separated by SDS-PAGE. The site-specific phosphorylation and expression of ZAP-70 was assessed by immunoblotting using ZAP-70 pY319 (top) and pan-ZAP-70 (bottom) antibodies. (B) The immunoblots were analyzed by densitometry and the ratio of the intensity of the phosphospecific ZAP-70 band to the pan-ZAP-70 band for each cell line was averaged. Letters represents significant differences of p<0.05.

Upon engagement of the TCR with a specific peptide-MHC complex, the tyrosine kinases ZAP-70 is phosphorylated and activated by the Src family kinases Lck and Fyn [1]. Therefore, it was of interest to determine if there were differences in the TCR-induced phosphorylation of ZAP-70 among Jurkat E6.1 T cells, HuT78 T cells and APBTs. Phosphorylation of ZAP-70 at tyrosine residue 319 is required for its activation and subsequent phosphorylation of the adaptor molecules LAT and SLP-76 [21], [22]. The phosphorylation of this tyrosine is an excellent read-out for enzymatically active ZAP-70. As seen in Figure 3, the relative levels of tyrosine 319 phosphorylation were similar between HuT78 T cells and APBTs. However, there was a significant and reproducible 2–3 fold decrease in the site-specific phosphorylation of ZAP-70 in Jurkat E6.1 T cells (Figure 3B). This suggests that Jurkat E6.1 T cells have reduced levels of TCR-induced ZAP-70 activation and potentially have altered downstream signaling.

**Figure 4:**
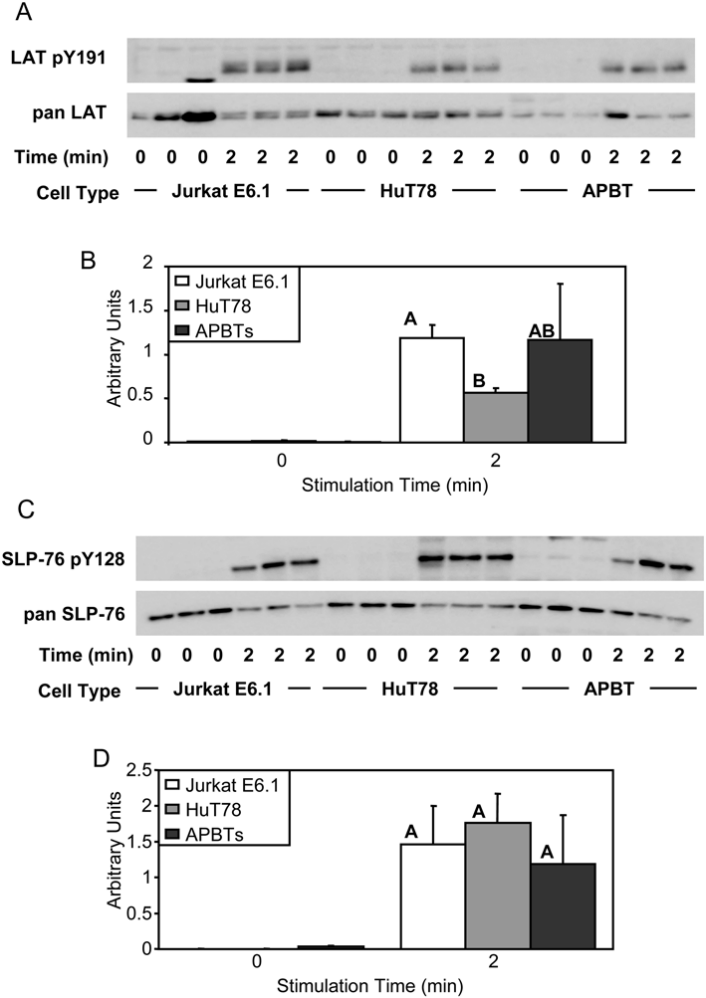
Jurkat E6.1 T cells, HuT78 T cells, and APBTs have similar levels of LAT and SLP-76 phosphorylation. (A) Three samples of Jurkat E6.1 T cells, HuT78 T cells, and APBTs were stimulated and the cellular proteins were separated by SDS-PAGE. The site-specific phosphorylation and expression of LAT was assessed by immunoblotting using LAT pY191 (top) and pan-LAT (bottom) antibodies. (B) The immunoblots were analyzed by densitometry and the ratio of the intensity of the phosphospecific LAT band to the pan-LAT band for each cell line was averaged. Letters represents significant differences of p<0.01. (C) Three samples of Jurkat E6.1 T cells, HuT78 T cells, and APBTs were stimulated and the cellular proteins were separated by SDS-PAGE. The site-specific phosphorylation and expression of SLP-76 was assessed by immunoblotting using SLP-76 pY128 (top) and pan-SLP-76 (bottom) antibodies. (D) The immunoblots were analyzed by densitometry and the ratio of the intensity of the phosphospecific SLP-76 band to the pan-SLP-76 band for each cell line was averaged. There were no significant differences between cell lines and p>0.25.

We next examined the phosphorylation of signaling proteins downstream of ZAP-70 to see if the diminished phosphorylation of this important early kinase translated to later differences in TCR-mediated signaling. ZAP-70 is the known kinase for the adaptor proteins LAT and SLP-76 [23]. The phosphorylation of LAT at tyrosine 191 is essential for the formation of multi-protein signaling complexes that transmit the signal from the TCR complex to downstream effectors [7], [8]. When the relative levels of LAT tyrosine 191 phosphorylation were examined, no differences were observed between Jurkat E6.1 and APBT samples or between HuT78 and APBT samples (Figure 4A and 4B). However, there was a small but significant difference in the phosphorylation of LAT tyrosine 191 between Jurkat E6.1 and HuT78 T cell lines (Figure 4A and 4B). These data show that the phosphorylation of LAT is not substantially different among the cell lines, indicating the binding capacity of LAT in each cell is similar.

Another important adaptor protein for functions downstream of TCR activation is SLP-76. This protein is phosphorylated on three tyrosine residues, including tyrosine 128. Phosphorylated SLP-76 is essential for bringing Vav1 and Itk to the LAT signaling complex [10], which is vital for linking proximal TCR signaling to events such as calcium influx and actin cytoskeletal rearrangement. As seen for LAT, no difference was seen among the two cell lines and APBTs in the phosphorylation of SLP-76 tyrosine 128 (Figure 4C and 4D), suggesting that SLP-76 has a similar signaling capacity in each type of cell. Together, these data indicate that the reduced levels of TCR α/β chain surface expression in the HuT78 T cells compared to other T cell lines and the decrease in ZAP-70 phosphorylation observed in Jurkat E6.1 T cells compared to HuT78 T cells and APBTs does not translate into differences in the activation of signaling proteins immediately downstream of ZAP-70.

### Jurkat E6.1 T cells have hyperphosphorylated PLC-γ1 and exhibited exaggerated Ca^2+^ signaling compared to HuT78 T cells and APBTs

**Figure 5:**
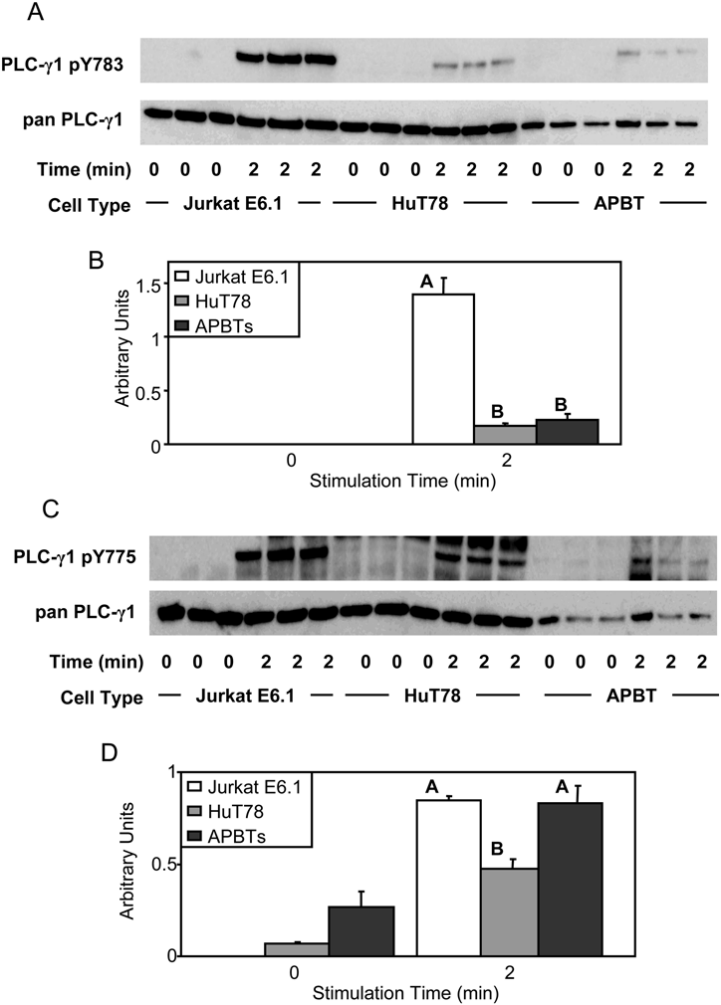
T cell lines have differences in PLCγ1 phosphorylation. (A) Three samples of Jurkat E6.1 T cells, HuT78 T cells, and APBTs were stimulated and the cellular proteins were separated by SDS-PAGE. The site-specific phosphorylation and expression of PLCγ1 was assessed by immunoblotting using PLCγ1 pY783 (top) and pan-PLCγ1 (bottom) antibodies. (B) The immunoblots were analyzed by densitometry and the ratio of the intensity of the phosphospecific PLC-γ1 band to the pan-PLC-γ1 band for each cell line was averaged. Letters represents significant differences of p<0.0005. (C) Three samples of Jurkat E6.1 T cells, HuT78 T cells, and APBTs were stimulated and the cellular proteins were separated by SDS-PAGE. The site-specific phosphorylation and expression of PLCγ1 was assessed by immunoblotting using PLCγ1 pY775 (top) and pan-PLCγ1 (bottom) antibodies. (D) The immunoblots were analyzed by densitometry and the ratio of the intensity of the phosphospecific PLC-γ1 band to the pan-PLC-γ1 band for each cell line was averaged. Letters represents significant differences of p<0.03.

Next, differences in several important signaling events downstream of LAT and SLP-76 were characterized in Jurkat E6.1 T cells, HuT78 T cells, and APBTs. PLC-γ1 activation is important for both intracellular calcium flux and protein kinase C activation [1], [8]. Tyrosine 783 on PLC-γ1 is phosphorylated by the Tec family kinase Itk and is essential for PLC-γ1 activation and enzymatic function [13], [24], [25]. Therefore, the phosphorylation of PLC-γ1 tyrosine 783 was characterized to determine if there was a difference in the phosphorylation of this important residue among the T cell lines. Surprisingly, a 10-fold increase in the relative phosphorylation of PLC-γ1 tyrosine 783 was observed in the Jurkat E6.1 T cell line, compared to the HuT78 T cells and APBTs (Figure 5A and 5B). In contrast, no difference in the site-specific phosphorylation of PLC-γ1 was found between the HuT78 T cells and APBTs (Figure 5A and 5B). This suggests that PLCγ1 is substantially more activated in Jurkat E6.1 T cells compared to HuT78 T cells or APBTs.

The phosphorylation of tyrosine 775 on PLCγ1 is also known to be critical for TCR-induced Ca^2+^ influx and transcription factor activation [26]. Therefore, we examined whether the TCR-mediated phosphorylation of this site is also increased in Jurkat E6.1 T cells compared to other T cell lines. Interestingly, Jurkat E6.1 T cells and APBTs had no detectable difference in the TCR-induced phosphorylation of tyrosine 775 on PLCγ1 (Figure 5C and 5D). In contrast, HuT78 T cells had significantly less phosphorylation of this site compared to both Jurkat E6.1 T cells and APBTs (Figure 5C and 5D). This indicates that the phosphorylation of tyrosines 775 and 783 on PLCγ1, which are both required for optimal TCR-induced function of this protein, may be differentially regulated.

**Figure 6:**
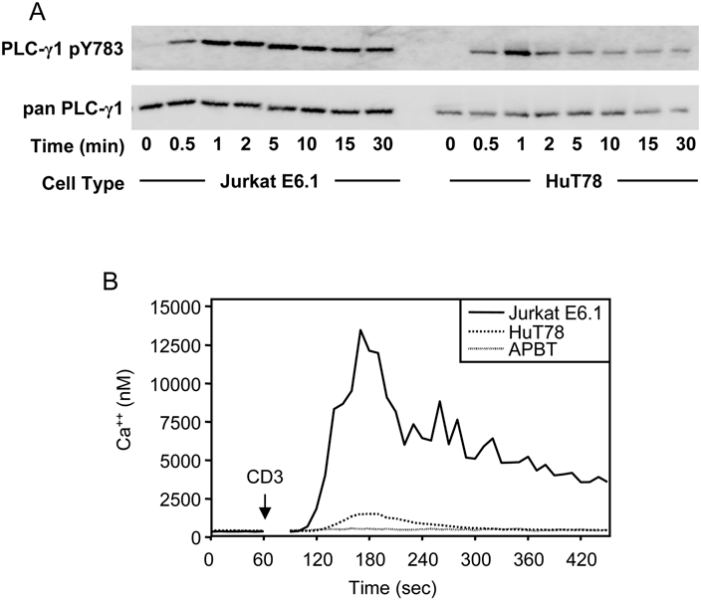
Jurkat E6.1 T cells have increased PLCγ1 function compared to HuT78 T cells and APBTs. (A) Jurkat E6.1 T cells and HuT78 T cells were stimulated over a 30′ time course and the cellular proteins were separated by SDS-PAGE. The site-specific phosphorylation and expression of PLCγ1 and the expression of actin was assessed by immunoblotting using PLCγ1 pY783 (top) and pan-PLCγ1 (bottom) antibodies. (B) TCR-induced calcium flux was measured quantitatively as described above. Traces for Jurkat E6.1 T cells, HuT78 T cells and APBTs are shown.

The observation that PLCγ1 Y783 is hyperphosphorylated in Jurkat E6.1 T cells compared to HuT78 T cells and APBTs lead us to further examine the kinetics of PLC-γ1 phosphorylation. This was done in order to determine if the large differences in phosphorylation were seen not only in the amount of TCR-induced phosphorylation of PLC-γ1 tyrosine 783 at a single timepoint, but also in the timing and duration of this phosphorylation event. A substantial difference was seen in the kinetics of phosphorylation of PLC-γ1 tyrosine 783 between Jurkat E6.1 T cells, and HuT78 T cells. HuT78 T cells reached maximal phosphorylation at 1 minute post-TCR stimulation, followed by a steady decrease in phosphorylation over 30 minutes (Figure 6A). APBTs and HuT78 T cells had similar TCR-induced phosphorylation kinetics of PLC-γ1 Y783 (data not shown). In contrast, the Jurkat E6.1 T cell line reached maximal phosphorylation at 2 minutes and the phosphorylation of tyrosine 783 decreased only slightly over the course of 30 minutes (Figure 6A). This indicates that Jurkat E6.1 T cell have not only greater relative PLC-γ1 tyrosine 783 phosphorylation than HuT78 T cells and APBTs, but that they also sustain that level of hyperphosphorylation for a much longer time.

The TCR-induced influx of Ca^2+^ into human T cells is controlled by PLC-γ1 activation. The influx of Ca^2+^ is important for many effectors functions in T cells, including NFAT activation and cytokine and chemokine production [1], [27]. Due to its importance in downstream T cell functions, we examined whether the hyperphosphorylation of PLC-γ1 in Jurkat E6.1 T cells lead to alterations in the levels of TCR-induced Ca^2+^ influx in the T cell lines and APBTs. To this end, we utilized a quantitative, real-time measurement method for intracellular Ca^2+^ developed by Tsien and coworkers [28]. Using this method, it was observed that both cell lines and APBTs showed TCR inducible Ca^2+^ influx (Figure 6B). Jurkat E6.1 T cells exhibited approximately 7–10-fold higher Ca^2+^ flux immediately following stimulation than HuT78 T cells or APBTs, as well as a higher level and longer time of sustained Ca^2+^ (Figure 6B). HuT78 T cells also showed higher levels of initial Ca^2+^ signaling than APBTs, although they returned to similar levels as APBTs much more quickly than Jurkat E6.1 T cells (Figure 6B). These data indicate that the increased phosphorylation of PLC-γ1 tyrosine 783 in Jurkat E6.1 T cells also translated into an amplification in Ca^2+^ flux, as compared to HuT78 and APBT cells.

### Jurkat E6.1 T cells have hyperphosphorylated Pyk2 and Vav1 compared to HuT78 T cells and APBTs

**Figure 7:**
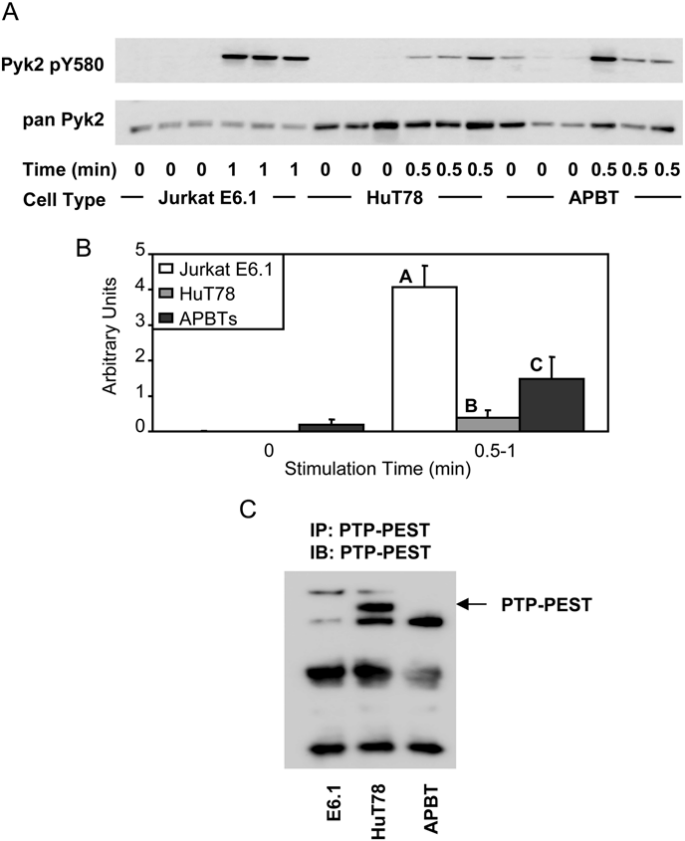
Jurkat E6.1 T cells, HuT78 T cells, and APBTs have different levels of Pyk2 Y580 phosphorylation. (A) Three samples of Jurkat E6.1 T cells, HuT78 T cells, and APBTs were stimulated and the cellular proteins were separated by SDS-PAGE. The site-specific phosphorylation and expression of Pyk2 was assessed by immunoblotting using Pyk2 pY580 (top) and pan-Pyk2 (bottom) antibodies. (B) The immunoblots were analyzed by densitometry and the ratio of the intensity of the phosphospecific Pyk2 band to the pan-Pyk2 band for each cell line was averaged. Letters represents significant differences of p<0.05. (C) PTP-PEST was immunoprecipitated for Jurkat E6.1 T cells, HuT78 T cells and APBTs. The expression of PTP-PEST in the different T cells was assessed by immunoblotting.

The intracellular tyrosine kinase Pyk2 is critical for integrating receptor mediated signals that control the rearrangement of the actin cytoskeleton [6]. The levels of the enzymatic activity of Pyk2 is controlled by the phosphorylation of tyrosine 580 in the activation loop of the kinase domain [6]. Therefore, the phosphorylation of this site can be used as a marker for the induction of the kinase activity of Pyk2. To investigate the relative activation of Pyk2 in Jurkat E6.1 T cells, HuT78 T cells, and APBTs, the cells were stimulated for 1 minute (Jurkat E6.1 cells) or 30 seconds (HuT78 T cells and APBTs), which corresponded to the maximal activation time of Pyk2 in these cell types (M. Collins and J.C.D. Houtman, unpublished results). Interestingly, the relative level of Pyk2 tyrosine 580 phosphorylation was significantly different between Jurkat E6.1 T cells, HuT78 T cells and APBTs, with Jurkat E6.1 T cells having the highest relative level of Pyk2 tyrosine 580 phosphorylation and HuT78 T cells having the lowest (Figure 7A and Figure 5B). These experiments show that the enzymatic activity of Pyk2 is different in the individual T cell lines, suggesting that there are differences in the ability of the TCR to control the actin cytoskeleton between Jurkat E6.1 T cells, HuT78 T cells and APBTs.

One potential mechanism for these differences is the differential expression of PTP-PEST, a tyrosine phosphatase known to dephosphorylate Pyk2 in T cells [29]. To investigate this possibility, the expression of PTP-PEST in the three T cell lines was characterized. Unfortunately, the expression of PTP-PEST was not detectable in cell lysates (data not shown). Therefore, PTP-PEST was immunoprecipitated from unstimulated Jurkat E6.1 T cells, HuT78 T cells and APBTs. As seen in Figure 7C, HuT78 T cells express PTP-PEST, whereas Jurkat E6.1 T cells and APBTs have no detectable expression of this phosphatase. These results agree with a recent study that also observed the Jurkat E6.1 T cells and APBTs do not express PTP-PEST [30]. These data suggest that the expression of PTP-PEST in HuT78 T cells results in reduced levels of Pyk2 phosphorylation compared to APBTs. In contrast, the significant differences in the TCR-induced phosphorylation of Pyk2 in Jurkat E6.1 T cells and APBTs are not due to differential expression of this phosphatase.

**Figure 8:**
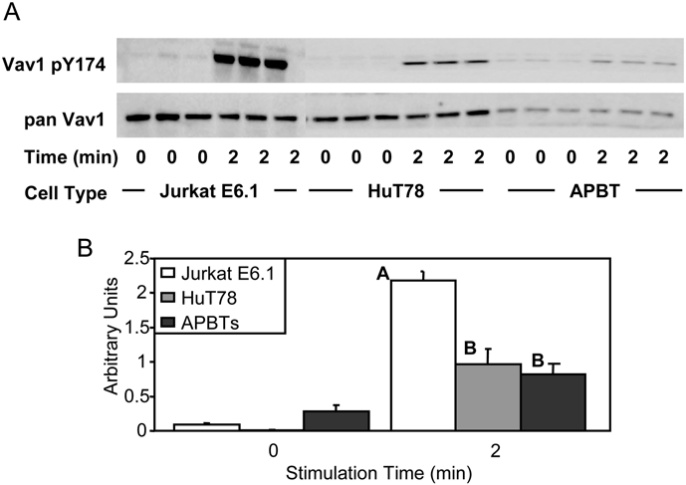
Jurkat E6.1 T cells exhibit hyperphosphorylated of Vav1 Y174 compared to HuT78 T cells and APBTs. (A) Three samples of Jurkat E6.1 T cells, HuT78 T cells, and APBTs were stimulated and the cellular proteins were separated by SDS-PAGE. The site-specific phosphorylation and expression of Vav1 was assessed by immunoblotting using Vav1 pY174 (top) and pan-Vav1 (bottom) antibodies. (B) The immunoblots were analyzed by densitometry and the ratio of the intensity of the phosphospecific Vav1 band to the pan-Vav1 band for each cell line was averaged. Letters represents significant differences of p<0.005.

Vav1 is a guanine nucleotide exchange factor for the small GTPase Rac1 and is important for receptor-mediated actin cytoskeletal rearrangement in human T cells [11]. An obligate step in the enzymatic activation of Vav1 is the phosphorylation of tyrosine 174 [11]. In contrast to Pyk2, Vav1 tyrosine 174 had a moderate 2-fold hyperphosphorylation in Jurkat E6.1 T cells compared to both HuT78 T cells and APBTs (Figure 8A and Figure 6B). These data suggest that Vav1 is hyperactivated in Jurkat E6.1 T cells, which, in conjunction with the hyperphosphorylation of Pyk2, indicates a potential distortion of cytoskeletal rearrangement in these cells.

### Itk, the common link between PLC-γ1 and Vav1, has increased expression in Jurkat E6.1 T cells

**Figure 9:**
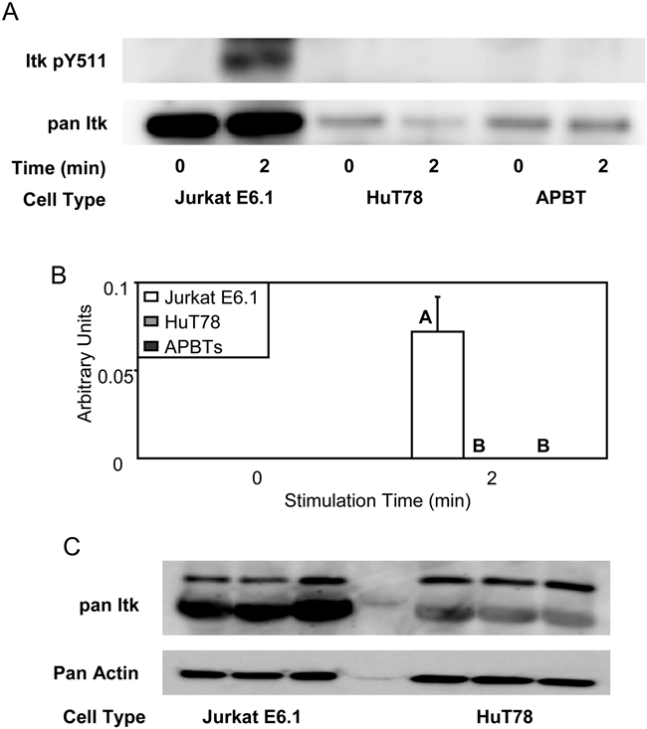
Jurkat E6.1 T cells have increased expression and TCR-induced phosphorylation of Itk compared to HuT78 T cells and APBTs. (A) Itk was immunoprecipitated from lysates for TCR-induced Jurkat E6.1 T cells, HuT78 T cells, and APBTs and the cellular proteins were separated by SDS-PAGE. The site-specific phosphorylation and expression of Itk was assessed by immunoblotting using Itk pY511 (top) and pan-Itk (bottom) antibodies. (B) The immunoblots were analyzed by densitometry and the ratio of the intensity of the phosphospecific Itk band to the pan-Itk band for each cell line for three separate experiments was averaged. Letters represents significant differences of p<0.04. (C) Whole cell lysates of Jurkat E6.1 T cells and HuT78 T cells were prepared and immunoblotted using pan-Itk (top) and actin (bottom) antibodies.

The hyperphosphorylation of both Vav1 and PLC-γ1 at important tyrosine residues, as well as exaggerated Ca^2+^ flux, in Jurkat E6.1 T cells led us to examine Itk, the common link among these events. Itk requires Vav1 to bind SLP-76, an event necessary for Itk to phosphorylate PLC-γ1 Y783 [12]. Phosphorylation of tyrosine residue 511 is required for the activation of Itk [31], therefore the phosphorylation of this site serves as a marker for the induction of Itk kinase activity. Unfortunately, we were unable to directly detect any TCR-induced site-specific phosphorylation of Itk in stimulated T cell lysates (data not shown). Consequently, Itk immunoprecipitations were performed to examine differences in the phosphorylation of Itk tyrosine 511. Interestingly, there was observable TCR-induced phosphorylation of Itk tyrosine 511in Jurkat E6.1 T cells, but little detectable phosphorylation of this site in HuT78 T cells and APBTs (Figure 9A and Figure 7B). In addition, there was an increase in the amount of immunoprecipitated Itk in Jurkat E6.1 T cells compared to HuT78 T cells and APBTs (Figure 9A), suggesting Jurkat E6.1 cells have increased expression of Itk. To more directly compare expression of Itk in the T cell lines, the levels of Itk in an equal cell number of Jurkat E6.1 T cells and HuT78 T cells, and APBTs was measured by immunoblotting. Jurkat E6.1 T cells had at least a 2-fold greater Itk expression than HuT78 T cells (Figure 9C). Jurkat E6.1 T cells from different laboratories had differences in the relative levels of Itk, with most having increased levels of this protein. This suggests that there are variations in the relative levels of signaling proteins in individual sublines of the Jurkat E6.1 T cells. Collectively, these data indicate that the increased expression of Itk in Jurkat E6.1 T cells compared to HuT78 T cells and APBTs is linked to exaggerated TCR-mediated signaling.

### Jurkat E6.1 T cells have hyperphosphorylated Erk1 and Erk2 compared to HuT78 T cells and APBTs

**Figure 10:**
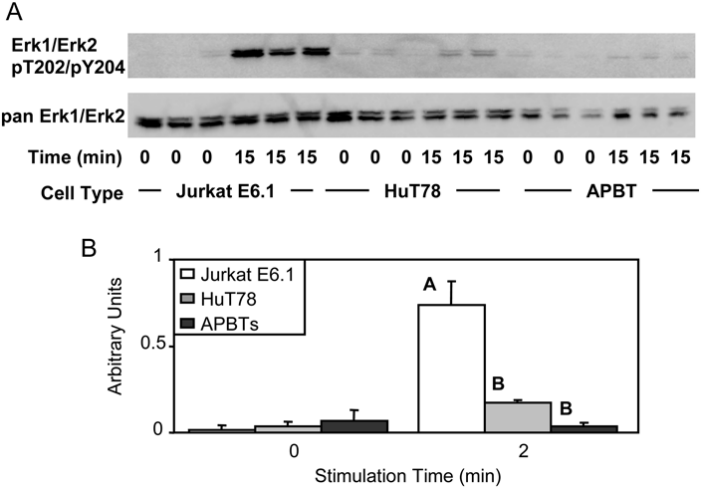
Jurkat E6.1 T cells have hyperphosphorylated Erk1/Erk2 compared to HuT78 T cells and APBTs. (A) Three samples of Jurkat E6.1 T cells, HuT78 T cells, and APBTs were stimulated and the cellular proteins were separated by SDS-PAGE. The site-specific phosphorylation and expression of Erk1 and Erk2 was assessed by immunoblotting using Erk1/Erk2 pT202/pY204 (top) and pan-Erk1/Erk2 (bottom) antibodies. (B) The immunoblots were analyzed by densitometry and the ratio of the intensity of the phosphospecific Erk1/Erk2 bands to the pan-Erk1/Erk2 bands for each cell line for three separate experiments was averaged. Letters represents significant differences of p<0.005.

The MAP kinases Erk1 and Erk2 are important for the regulation of the expression and function of the transcription factor AP-1 [1]. The activation of these kinases is downstream of LAT phosphorylation, Vav1 activation, and Ca^2+^ influx [1]. The phosphorylation of Erk1 and Erk2 on threonine 202 and tyrosine 204 in the activation loop of their kinase domain is required for full enzymatic activity of these proteins. Thus, the dual phosphorylation of these sites is a measure of the enzymatic activity of these kinases. Due to its important role in linking cytoplasmic signaling events to changes in gene transcription, the phosphorylation of Erk1 and Erk2 was determined in the three T cell lines. A significant increase in the TCR-induced phosphorylation of Erk1 and Erk2 was observed in Jurkat E6.1 T cells compared to HuT78 T cells and APBTs (Figure 10A and 10B). This is consistent with the increase in the phosphorylation of other important signaling molecules in Jurkat E6.1 cells and indicates that these T cells have exaggerated distal signaling events compared to HuT78 T cells and APBTs.

### Jurkat E6.1 T cells, HuT78 T cells, and APBTs have distinct patterns of TCR-induced cytokine release and costimulatory receptor expression and function

TCR activation leads to the increased production and release of numerous cytokines and chemokines that affect the function and localization of immune cell types. Because of the importance of cytokines and chemokines in the host immune response, we examined the release of these molecules from Jurkat E6.1 T cells, HuT78 T cells and APBTs upon TCR activation. For these studies, the T cell lines were stimulated for 24 hours using plate-bound stimulatory anti-TCR antibody and the release of 28 chemokines and cytokines was quantitatively measured using a cytokine multiplex assay. As seen in Table 1, APBTs released a wide range of cytokines and chemokines upon TCR stimulation. These include Th1- and Th2-associated cytokines and chemokines and the Th17 associated cytokine, IL-17 (Table 1) [32], [33], [34], [35]. The release of such a wide range of cytokines and chemokines was due to the varied nature of the APBTs, which contain mixture of CD4+ and CD8+ T cells. In contrast, Jurkat E6.1 T cells release a more limited pool of cytokines and chemokines, with only IL-2 and the chemokines, IL-8, MIP-1α, MIP-1β and MCP-1 being produced in detectable quantities (Table 1). HuT78 T cells released a wider range of chemokines and cytokines than Jurkat E6.1 T cells, including a mixture of Th1-and Th-2 associated chemokines and cytokines. These data suggest that HuT78 T cells are more similar to APBTs than Jurkat E6.1 T cells in their TCR-induced production of cytokines and chemokines.

**Table 1:** TCR-induced release of cytokines and chemokines from Jurkat E6.1 T cells, HuT78 T cell, and APBTs.

Cytokine	Jurkat E6.1	HuT78	APBT
IL-1β	-	-	-
IL-2	+++	+++	+/++
IL-4	-	-	+
IL-5	-	-	+
IL-6	-	-	-
IL-7	-	-	++
IL-8/CXCL8	+++	-	+/++
IL-10	-	+++	+/++
IL-12	-	+	+
IL-13	-	+	+/++
IL-15	-	-	-
IL-17	-	-	+
VEGF	-	++	++
TNF-α	-	-	++
IFN-α	-	-	-/+
IFN-γ	-	-	++
GM-CSF	-	++	++/+++
MIP-1α/CCL3	++	+	+++
MIP-1β/CCL4	++	++	+++
IP-10/CXCL10	-	+	-
MIG/CXCL9	-	+	+
Eotaxin/CCL11	-	-	-
RANTES/CCL5	-	+++	++/+++
MCP-1/CCL2	++	-	++
G-CSF	-	++	+/++
EGF	-	-	-
Basic FGF	-	+	+
HGF	-	-	-

- no detectable release.

+<100 pg/mL.

++ 101–999 pg/mL.

+++>1000 pg/mL.

**Figure 11:**
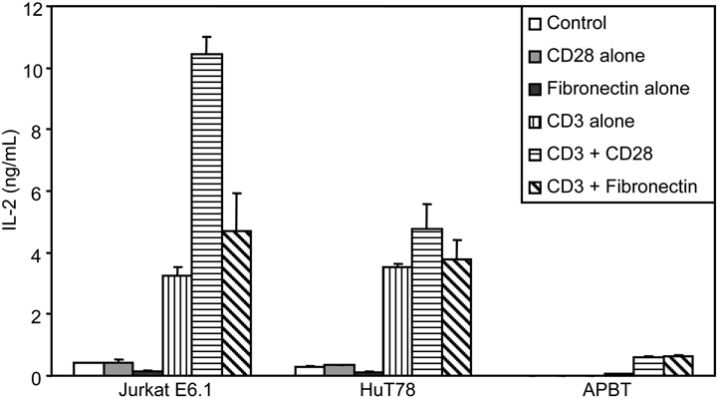
Jurkat E6.1 T cells and HuT78 T cells have aberrant IL-2 production compared to APBTs. Jurkat E6.1 T cells, HuT78 T cells and APBTs were stimulated using a CD3 antibody (1 µg/mL), both alone and in combination with a stimulatory CD28 antibody (2 µg/mL) and fibronectin (1 µg/mL). The production of IL-2 after 24 hours of stimulation was assessed by ELISA as described above.

One important cytokine produced by TCR-stimulated T cells is IL-2, which controls T cell growth and differentiation [34]. As IL-2 production is considered an excellent way to measure cumulative activation of a T cell and it was the only cytokine produced by all three cell lines, we decided to examine the production of IL-2 by TCR-stimulated Jurkat E6.1 T cells, HuT78 T cells, and APBTs. For these studies, the T cell lines were stimulated with plate-bound stimulatory TCR antibody alone or in combination with soluble stimulatory CD28 antibody or plate-bound fibronectin, the ligand for VLA-4. These activators were chosen to investigate the coordinate induction of two well studied costimulatory receptors, CD28 and the VLA-4 integrin, and the TCR. To this end, APBTs produced IL-2 when stimulated with TCR antibody, but co-treatment with either CD28 antibody or fibronectin lead to a significant 10×–12×-fold increase in IL-2 production (Figure 11). There was no significant difference between the IL-2 production in APBTs costimulated with either the CD28 antibody or fibronectin (Figure 11). Jurkat E6.1 T cells also produced IL-2 in response to TCR antibody treatment and had approximately 3×-fold more IL-2 production upon costimulation with the CD28 antibody (Figure 11). In contrast, costimulation of Jurkat E6.1 T cells with fibronectin resulted in a small, but not significant, increase in IL-2 production over TCR stimulation alone. HuT78 T cells showed no statistically significant increase in IL-2 production when co-treated with the CD28 antibody or fibronectin compared to TCR antibody alone (Figure 11). It is of note, however, that both Jurkat E6.1 T cells and HuT78 T cells consistently produced substantially more IL-2 than APBTs upon treatment with similar concentrations of TCR antibody alone, suggesting both cell lines have increased expression of IL-2 upon TCR activation.

To further investigate the surprising unresponsiveness of Jurkat E6.1 and HuT78 T cells to CD28 and/or VLA-4-mediated signals, we characterized the expression of several critical cell surface receptors on Jurkat E6.1 T cells, HuT78 T cells, and APBTs by flow cytometry. As expected, all three cell lines had high expression of the TCR (Table 2). In contrast, there was variable expression of the co-receptor CD4. Jurkat E6.1 T cells had little to no detectable CD4 expression whereas the majority of HuT78 T cells had a low but detectable expression of this protein (Table 2). APBTs had substantial percentage of cells that expressed CD4, with levels between 50%–80% depending on the donor (Table 2). All three cells lines had high surface levels of the costimulatory receptor CD7 and the integrins LFA-1 and VLA-4 (Table 2). Interestingly, Jurkat E6.1 T cells and APBTs also had substantial expression of the costimulatory receptors CD28 and CD2, whereas HuT78 T cells had no detectable expression of these molecules (Table 2). APBTs expressed both the activation-induced receptors CD44 and CD137 (Table 2), as expected since these cells were stimulated upon expansion. In contrast, Jurkat E6.1 cells had no detectable levels of either CD44 or CD137 (Table 2). Interestingly, HuT78 T cells had low but detectable levels of CD44 but did not expression CD137 in the unactivated state (Table 2). However, TCR stimulation could upregulated CD137 in HuT78 T cells but not in Jurkat E6.1 T cells (data not shown). These data show that Jurkat E6.1 T cells, HuT78 T cells and APBTs have different patterns of costimulatory receptor expression, indicating that Jurkat E6.1 and HuT T cells would be distinctly useful for the study of costimulatory receptors. In addition, these data suggest that the unresponsiveness of HuT78 T cells to CD28 stimulatory signals is due the lack of surface expression of CD28. The reason for the unresponsiveness of Jurkat E6.1 and HuT78 T cells to fibronectin is unknown, since both cell lines express VLA-4 on their surface.

**Table 2:** Expression of surface receptors in Jurkat E6.1 T cells, HuT78 T cell, and APBTs.

Receptor	Jurkat E6.1	HuT78	APBT
TCR	+++	+++	+++
CD4	-	++	++/+++
CD28	+++	-	+++
CD2	+++	-	+++
CD7	+++	+++	+++
CD11a/LFA-1	+++	+++	+++
CD49d/VLA-4	+++	+++	+++
CD44	-	+	+
CD137/4-1BB	-	-	+

+++>75%.

++50–75%.

+25–50%.

-<25%.

## Discussion

The inappropriate activation or suppression of T cells can be implicated in nearly all human diseases, including cancer, heart disease, multiple sclerosis, asthma, allergies, diabetes, and many others [3], [4], [36], [37], [38]. Early signaling pathways induced by both the TCR and/or costimulatory receptors are excellent targets for therapies to these diseases. Although a great deal is known about the induction of signaling by these receptors, much more must be learned before therapies can be effectively developed to target these pathways. T cell lines are an integral part of the research of T cell signaling due to their ease of use, low cost, and reduced human burden. However, not all cell lines are equally useful as a model system of human T cells. In this paper, we investigated differences in peripheral TCR signaling, cytokine release and costimulatory receptor function and expression among the Jurkat E6.1 T cell line, the HuT78 T cell line, and APBTs.

To examine the relative extent of activation of key signaling molecules in Jurkat E6.1 T cells, HuT78 T cells, and APBTs, we used a previously described method for quantifying the amount of phosphorylation per protein present in an activated T cell [24], [39]. By using this method, the total phosphorylation per protein of a specific signaling molecule for each cell type was determined and compared, regardless of the total amount of protein present. This is important since there were substantial differences between the T cell lines and APBTS in the expression of several of the examined signaling proteins, including ZAP-70, Pyk2 and Vav1. By normalizing the phosphorylation level of each protein to the absolute amount expressed in each cell type, we were able to eliminate potential artifacts due to differences in expression levels. 

The first signaling event to occur upon TCR activation is the stimulation of the Src family kinases Lck and Fyn [1]. We investigated the phosphorylation of Lck and Fyn at the activating tyrosine residue 394 and 420, respectively, and found that these sites had constitutive phosphorylation in Jurkat E6.1 T cells, but moderate TCR-inducible phosphorylation in HuT78s and APBTs (data not shown). However, the low level of inducible phosphorylation of Lck and Fyn made the immunoblots difficult to quantify using the described method. These differences in the regulation of Lck and Fyn did not appear to matter since only a slight decrease was seen in the phosphorylation of tyrosine 319 on the tyrosine kinase ZAP-70 in Jurkat E6.1 T cells compared to HuT78 T cells and APBTs (Figure 3). This difference in ZAP-70 activation did not translate into changes in the phosphorylation of the ZAP-70 substrates, LAT and SLP-76 (Figure 4). Together, these results indicate that although there are small differences in events immediately following TCR activation, the relative levels of activated LAT and SLP-76 in each cell type remains the same.

The similarities between Jurkat E6.1 T cells, HuT78 T cells and APBTs ended at signaling events downstream of LAT and SLP-76. Jurkat E6.1 T cells have deficiencies in the expression of lipid phosphatases PTEN and SHIP, leading to the constitutive activation of AKT, a serine-threonine kinase important for cell survival, and other downstream signaling molecules [16], [17], [18]. In addition to this defect, we observed three new defects in Jurkat E6.1 T cells compared to APBTs. First, Jurkat E6.1 cells have a significant increase in the relative phosphorylation of PLCγ1 tyrosine 783 compared to HuT78 T cells and APBTs (Figure 5). In contrast, HuT78 T cells had reduced levels of PLCγ1 tyrosine 775 phosphorylation compared to Jurkat E6.1 and APBTs (Figure 5). Interestingly, these data indicate these two sites on PLCγ1 may have differential regulation by intracellular kinases and phosphatases. The end result of alterations in PLCγ1 phosphorylation in the various cell lines was that Jurkat E6.1 T cells exhibited a nearly 7×-fold greater TCR-induced Ca^2+^ flux than HuT78 T cells and APBTs (Figure 6). In addition, Jurkat E6.1 T cells also had increased levels of site-specific phosphorylation of Vav1 and Erk1/Erk2 (Figure 8 and Figure 10).

The reason for the distorted phosphorylation of PLCγ1 and Vav1 does not appear to be due to deficiencies in the expression of PTEN and SHIP, since we have recently shown that inhibition of PI3K does not alter the site specific phosphorylation of these proteins (NCO and JCDH, manuscript submitted). The hyperphosphorylation of Erk1/Erk2 and the increased levels of TCR-induced Ca^2+^ influx could, in part, be due to decreased expression of PTEN and SHIP, because PI3K signaling is needed for the optimal activation of these events in human T cells (NCO and JCDH, manuscript submitted). However, a probable mechanism for this distorted signaling appears to involve Itk, which had both higher levels of expression and phosphorylation at tyrosine 511 in Jurkat E6.1 T cells than in HuT78 T cells and APBTs (Figure 9). There was some variation in the Itk expression levels in various sublines of Jurkat E6.1 T cells, with most expressing high levels of Itk (data not shown). This indicates that genetic drift in the various sublines of this T cell line leads to differences in the expression of intracellular signaling proteins. Importantly, this is the first time that the over-expression of Itk, an crucial molecule at the intersection of TCR and costimulatory receptor-mediated signaling in T cells [40], has been observed in Jurkat E6.1 cells.

Next, we found that Pyk2 was hyperphosphorylated in TCR-stimulated Jurkat E6.1 T cells compared to HuT78 T cells and APBTs (Figure 7). Again, this is the first description of the increased phosphorylation of Pyk2 in Jurkat E6.1 T cells. The mechanism for the increased phosphorylation of Pyk2 is unknown, but does not appear to be due to alterations in the expression of PTP-PEST. Pyk2 is important since it integrates receptor signals controlling actin cytoskeletal rearrangement [6]. The increased relative phosphorylation of Pyk2, in conjunction with the augmented phosphorylation of Vav1 (Figure 8), suggests that the actin cytoskeletal rearrangement in Jurkat E6.1 T cells may be altered compared to HuT78 T cells and APBTs.

Finally, Jurkat E6.1 T cells release substantially fewer cytokines and chemokines upon TCR activation than HuT78 T cells and APBTs (Table 1). However, the amount of IL-2 and IL-8 released by Jurkat E6.1 T cells is substantially greater than the levels of these hormones produced by APBTs. These differences are likely due to the thymocytic origin of the Jurkat E6.1 T cells and the fact that the cells were selected for their ability to produce large amounts of IL-2 [14].

Although this study suggests that Jurkat E6.1 T cells differ in signaling from APBTs, they do have advantages (Table 3). The cells are incredibly resilient and easy to grow and maintain. There are also many useful mutant lines of Jurkat E6.1 T cells, each lacking a specific molecule in the peripheral TCR signaling pathway. Jurkat E6.1 T cells are remarkably easy to transfect, with transfection efficiencies routinely reaching 80–90%. Finally, a hidden advantage pointed to in this study is that they have exaggerated signaling, making changes much easier to detect. Even with the increased proximal signaling, studies using Jurkat E6.1 are likely valid since many of the discoveries and observations initially found in Jurkat E6.1 T cells have been subsequently shown in APBTs. Together, the data show that although the Jurkat E6.1 T cell line has substantially altered TCR-induced signaling, this cell line can still be a useful model for TCR signaling if deficiencies and problems are taken into account and the results are confirmed in APBTs.

**Table 3:** Phosphorylation per protein in Jurkat E6.1 and HuT78 cell lines as compared to APBTs.

Protein	Jurkat E6.1	HuT78
ZAP-70 Y319	<	=
LAT Y191	=	=
SLP-76 Y128	=	=
PLCγ1 Y783	>	=
PLCγ1 Y775	=	<
Vav1 Y174	>	=
Pyk2 Y580	>	<
Erk 1/2 Y	>	=

< indicates significantly less phosphorylation per protein than APBTs.

> indicates significantly more phosphorylation per protein than APBTs.

=  indicates no significant difference in phosphorylation per protein as compared to APBTs.

Conversely, this study points to the many similarities between HuT78 T cells and APBTs. One significant difference in the relative levels of early TCR-induced signaling events between HuT78 T cells and APBTs was the reduced TCR-induced phosphorylation of Pyk2 in HuT78 T cells (Figure 7). This is likely due to the expression of PTP-PEST, a known phosphatase for Pyk2, in HuT78 T cells and not activated APBTs. However, this difference may be less important than it seems, since a recent study has shown that PTP-PEST is expressed in naïve primary T cells and not activated T cells, the subtype of APBTs used for these studies [30]. In addition, HuT78 T cells produce a greater number of cytokines and chemokines upon TCR stimulation than Jurkat E6.1 T cells. Finally, HuT78 T cell can express several costimulatory receptors found in activated T cells, allowing investigation of these costimulatory pathways in a model T cell line. HuT78 T cells also have disadvantages, however. They do not express the costimulatory receptors CD2 or CD28 (Table 2), and are unable to be co-stimulated by fibronectin, suggesting that although VLA-4 is expressed, it may not be functional in these cells (Figure 11). In addition, HuT78 cells have elevated levels of IL-2 production without costimulation as compared to APBTs (Figure 11). HuT78 T cells also have abnormal c-myc function and over express Bcl-xL, which indicates possible problems with the anti-apoptotic pathway in these cells [19], [20]. All of this information must be kept in mind when choosing to use HuT78 T cells as a model system for T cell biology.

The results of this study indicate that HuT78 T cells are similar to APBTs in peripheral TCR signaling, whereas Jurkat E6.1 T cells exhibit exaggerated signaling (Table 3). In addition, there were differences in cytokine release and costimulatory receptor expression and function between the various cell lines. These results are significant in that, for the first time, a direct comparison of commonly used cell lines and APBTs has been performed. Though it is clear that APBTs are the ideal cells to use for examining TCR signaling, this is not always feasible. Both Jurkat E6.1 T cells and HuT78 T cells have advantages and disadvantages, but HuT78 T cells are clearly the most like APBTs in the phosphorylation of important peripheral TCR signaling molecules and cytokine release. The relative advantages and disadvantages of each cell line must be taken into account when choosing a model cell line for individual experiments.

## Materials and Methods

### Ethics statement

This study was conducted according to the principles expressed in the Declaration of Helsinki. The study was approved by the Institutional Review Board of University of Iowa. All donors provided written informed consent for the collection of samples and subsequent analysis.

### Materials

RPMI 1640, Iscoves Modified Dulbecco's Media (IMDM), L-Glutamine, Penicillin-streptomycin, and PBS were purchased from Gibco. Fetal Bovine Serum (FBS) was acquired from Atlantic. Src family kinase pY416, pan-Lck, ZAP-70 pY319 (65E4), pan-ZAP (99F2), p44/42 MAPK and pan-PLCγ1 antibodies were bought from Cell Signaling Technology. LAT pY191, pan-LAT, mouse IgG, and actin antibodies were obtained from Millipore. SLP-76 pY128, Itk pY511, TCR α chain and CD4 antibodies were purchased from BD Pharmingen. Pan-Vav1 (C-14), all secondary antibodies, and protein A/G agarose beads were acquired form Santa Cruz Biotechnologies, Inc. The PLCγ pY783 and Erk1/Erk2 pY1857/187 antibodies and the human cytokine 30-plex luminex kit were bought from Biosource/Invitrogen. The PLCγ pY775 antibody was a gift of Dr. Barbara Rellahan. The pan-SLP-76 antibody was obtained from Antibody Solutions. The Vav1 pY174 antibody was purchased from Abcam, Inc. The pan-Itk antibody was acquired from Chemicon International. OKT3 was produced from hybridoma. CD2, CD4, CD7, CD11a, CD28, CD44, CD49d and CD137 antibodies were purchased from Biolegend. The pan human IL-2 antibodies were obtained from Ebiosciences. IL-2 was obtained from R&D Systems. Criterion Precast polyacramide gels (4–15% and 15% Tris-HCl) were purchased from Biorad. Immobilon PVDF Transfer Membrane was acquired from Millipore. Indo-1-AM was purchased from Invitrogen. All chemicals used were research grade and purchased from various sources.

### Cell line stimulation

The Jurkat E6.1 T cells were a kind gift of Dr. Lawrence Samelson. Jurkat E6.1 T cells were grown at 37°C in 5% CO_2_ using complete RPMI 1640 media (RPMI 1640 supplemented with 10% FBS, 2 mM L-glutamine, 50 U/mL penicillin, and 50 µg/mL streptomycin). HuT78 T cells were purchased from ATCC. HuT78 T cells were grown in IMDM media supplemented with 10% FBS, 2 mM L-glutamine, 50 U/mL penicillin, and 50 µg/mL streptomycin. Both cell lines were grown to a concentration of 2–5×10^5^ cells/mL. Cells were washed with RPMI 1640 before use and then resuspended at a density of 5×10^7^ cells/mL. Each sample of 4×10^6^ cells was then treated with 10 µg/mL CD3 antibody (OKT3) for various times. Samples were lysed with 4-fold excess hot 2× sample buffer (20 mM Tris pH 8.0, 2 mM EDTA, 2 mM Na_3_VO_4_, 20 mM DDT, 2% SDS, and 20% glycerol), heated at 95°C for 4 minutes, and sonicated to reduce viscosity.

### APBT isolation and stimulation

All the studies described below were approved by the Human Subjects Institutional Review Board at the University of Iowa. Healthy blood donors were recruited by word-of-mouth from the students and staff of biomedical laboratories at the Carver College of Medicine at the University of Iowa. Informed consent was obtained from all donors before commencement of blood draws. Peripheral blood T cells were isolated from the venous blood of healthy human donors as previously described [24]. The cells were then grown in culture for 5–7 days in complete RPMI 1640 media supplemented with 10 ng/mL IL-2. After incubation, the T cells were >90% positive for TCR α/β chains and >97% positive for CD28, CD2, CD7, CD11a and CD49d. Depending on the donor, the T cells were 30–70% positive for CD4, with the typical donor having ∼65% CD4 positive T cells.

To stimulate the cells, the T cells were washed with RPMI 1640 and resuspended to a concentration of 1×10^8^ cells/mL. After a short incubation on ice, cells were treated with 10 µg/mL CD3 (OKT3) and 10 µg/mL CD4 antibodies and returned to ice for 30 minutes. Both CD3 and CD4 antibodies were used since only concurrent stimulation with these antibodies, and not CD3 antibody treatment alone, could induce detectable TCR-mediated signaling [24]. The cells were warmed at 37°C for 10 minutes and immediately stimulated with 25 µg/mL mouse specific goat IgG antibody for various times. Samples were lysed with a 2-fold excess of hot 2× sample buffer, heated for 4 minutes at 95°C, and sonicated to reduce viscosity.

### Immunoblotting

The protein components of 2×10^5^ cell equivalents/ sample were separated by SDS-PAGE using 4–15% or 15% Criterion pre-cast polyacrylamide gels. The separated proteins were then transferred to PVDF and blocked with 5% milk in TBST for 45 minutes at room temperature. The membranes were then incubated at room temperature with primary antibody diluted in 5% milk for 1 hour, followed by the appropriate secondary antibody diluted in 5% milk for 30 minutes. The immunoblots were then visualized by chemiluminescence using a Fuji imager.

### Analysis of immunoblotting

Immunoblots were analyzed using NIH Image J. The ratio of phosphospecific to pan expression of each protein was determined for stimulated and unstimulated samples in each cell type, allowing the determination of the phosphorylation per protein in each cell. Stimulated sample ratios for each cell type were then averaged and compared to the other cell types using a two-tailed t-test assuming equal variance. Whole cell lysate samples used to compare Itk expression were also analyzed using NIH Image J and normalized using actin expression. The ratio of protein: actin was compared between cell lines using a two-tailed t-test assuming equal variance.

### Immunoprecipitations

Jurkat E6.1 T cells, HuT78 T cells, and APBTs were grown as indicated above. The cells were washed in RPMI 1640 and resuspended to a concentration of 5×10^7^ cells/mL. 100 µL of cells/sample were stimulated as described above for various times and lysed in 400 µL lysis buffer (25 mM TRIS pH 8.0, 150 mM NaCl, 1% Brij 97, 0.5% n-Octyl-beta-D-glucopyranoside, 5 mM EDTA, 1 mM Na_3_VO_4_, and protease inhibitor). Samples were incubated on ice with mixing for 20 minutes and centrifuged. Supernatant was added to 20 µL protein A/G agarose beads and rotated at 4°C for 30 minutes. Beads were pelleted and the supernatant was applied to 25 µL protein A/G agarose beads and 2 µg anti-Itk antibody and rotated at 4°C overnight. Pelleted beads were washed 4 times with 800 µL lysate buffer (25 mMTRIS pH 8.0, 150 mM NaCl, 1% Brij 97, and 0.5% octyl glucoside) and then the bound proteins were eluted with 30 µL hot 2× sample buffer. Samples were heated at 95°C for 4 minutes.

### Calcium influx

Calcium influx was measure quantitatively as previously described [34], [35]. Briefly, Jurkat E6.1, HuT78, and APBTs were grown as indicated above. The cells were washed in RPMI 1640 without phenol red and resuspended to a concentration of 1×10^6^ cells/mL in 1 mL RPMI 1640 without phenol red, 1 mM probenecid, and 5 µM indo-1-AM. The cells were incubated for 1 hour at 37°C. After incubation the cells were washed twice with RPMI 1640 without phenol red. The pellet was then resuspended in RPMI 1640 without phenol red and 1 mM probenecid. The cells were kept on ice and in the dark until use.

Measurements were done at 37°C with constant stirring in acrylic cuvettes using a Fluorolog fluorimeter. The cells were excited at 350 nm and emission was measured at λ_1_ = 405 nm and λ_2_ = 485 nm. Fluorescence at λ_1_ and λ_2_ was measured in unstimulated cells every 10 seconds for a total of 60 seconds. The cells were then stimulated with 10 µg/mL CD3 antibody and fluorescence at λ_1_ and λ_2_ measured every 10 seconds for a total of 360 seconds. Cells were then lysed with 0.1% Triton x-100 and florescence was measured at both λ_1_ and λ_2_. 2 mM EGTA was then added to sequester the Ca^2+^ from the dye and again, fluorescence was measured at both emission wavelengths. Ca^2+^ influx was then determined using the equation [Ca^2+^] = *K*_D_[(*R*−*R_min_*)/*R_max_−R*)](*S*_f2_/*S*_b2_), where *K*_D_ is the dissociation constant for indo-1 and Ca^2+^, *R* is the ratio of λ_1_ and λ_2_ at a specific time, *R_max_* is the ratio of λ_1_ and λ_2_ after the cells are lysed with 0.1% Triton x-100, *R_min_* is the ratio of λ_1_ to λ_2_ after treatment with 2 mM EGTA, and *S_f2_/S_b2_* is the ratio of free to bound dye as measured at λ_2_ [34], [35].

### Cytokine and chemokine measurements

Jurkat E6.1 T cells, HuT78 T cells, and APBTs were grown as indicated above. The cells were washed in RPMI 1640 and resuspended in complete RPMI 1640 media to a concentration of 1×10^6^ cells/mL. 0.5×10^6^ cells were then added to 24 well tissue culture plates containing 2 µg/mL plate-bound OKT3 in the absence or presence of 2 µg/mL soluble CD28 antibody or 1 µg/mL human fibronectin. The cells were then incubated for 24 hours at 37°C in 5% CO_2_.

The presence of cytokines and chemokines in the media were assessed using a human cytokine 30-plex luminex assay. Briefly, a mixture of fluorescent beads, each containing an antibody to one of 30 different human cytokines or chemokines, were incubated with standards of known quantities of each cytokine and chemokine or supernatants from treated cells for two hours at RT. A mixture of biotinylated antibodies to each cytokine or chemokine was added to the beads for 1 hour at RT and then streptavidin labeled with the fluorescent dye RPE was added for 30 minutes at RT. The amount of bound streptavidin to each type of fluorescent bead was then assessed using Bio-Rad Bio-Plex system.

The presence of IL-2 in the media was determined by standard sandwich ELISA. Briefly, a monoclonal IL-2 antibody was coated onto a RIA 96-well plate overnight at 4°C. Triplicates of IL-2 standards and supernatants from treated cells were then added to the plate and incubated overnight at 4°C. A biotinylated polyclonal IL-2 antibody was added to the plate for 2 hours at RT and then streptavidin-horse radish peroxidase (HRP) was added for 1 hour at RT. The amount of bound streptavidin was then assessed using TMB peroxidase that was acidified using 0.67 N H_2_SO_4_. The absorbance of each well at 450 nM was then measured using spectrophotometric plate reader.

### Flow cytometry

Jurkat E6.1 T cells, HuT78 T cells, and APBTs were grown as indicated above. The cells were washed in FACS Buffer (PBS with 10% fetal calf serum and 0.05% sodium azide) and resuspended in FACS Buffer to a concentration of 1×10^6^ cells/mL. 0.5×10^6^ cells were then incubated with isotype control or indicated primary antibodies for 30 minutes on ice. The cells were washed in FACS Buffer and again resuspended in FACS Buffer to a concentration of 1×10^6^ cells/mL. The cells were then incubated with a PE-labeled mouse IgG antibody for 30 minutes on ice. This step was eliminated for the TCR α chain, CD3 and CD28 antibodies, which were all directly conjugated to fluorescent dyes. The cells were washed in FACS Buffer and again resuspended in FACS Buffer to a concentration of 1×10^6^ cells/mL. The amount of primary and secondary antibody bound to each cell was these examined using a Becton Dickinson FACScan flow cytometer. The flow cytometry data was then analyzed using the FloJo analysis program. The percentage of positive cells were identified by determining the percentage of cells with fluorescence >98% of the isotype control. Mean fluorescence intensity was determined using FloJo analysis program. The differences in TCR surface expression was statistically compared between cell lines using a two-tailed t-test assuming equal variance.
